# Study on Similar Materials for Weakly Cemented Medium and Indoor Excavation Test

**DOI:** 10.3390/ma18132948

**Published:** 2025-06-22

**Authors:** Shanchao Hu, Lei Yang, Shihao Guo, Chenxi Zhang, Dawang Yin, Jinhao Dou, Yafei Cheng

**Affiliations:** 1 College of Energy and Mining Engineering, Shandong University of Science and Technology, Qingdao 266590, China; skd994527@sdust.edu.cn (S.H.); 15564801327@163.com (L.Y.); m15285817719@163.com (D.Y.); djh15092318906@163.com (J.D.); chengyafei911@163.com (Y.C.); 2 College of Water Resource & Hydropower, Sichuan University, Chengdu 610065, China; cpu18135835371@163.com

**Keywords:** weakly cemented medium, creep, similar material, model test

## Abstract

The escalating disasters caused by the movement of shallow buried strata in China’s western mining areas are increasingly threatening operational safety. A critical issue in ensuring secure mining practices in these areas is the creep failure of weakly cemented soft rock under low-stress conditions. The unique particle contact mechanisms in weakly cemented mudstone, combined with the persistence of the cemented materials and the particulate matter they form, lead to mechanical responses that differ significantly from those of typical soft rocks during loading. Building on an existing multivariate linear regression equation for new similar materials, this study developed qualified weakly cemented medium similar materials, offering appropriate materials for long-term creep tests of weakly cemented formations. This was accomplished by employing orthogonal proportioning tests. The principal findings of our investigation are as follows: The new, similar material exhibits low strength and prominent creep characteristics, accurately simulating weakly cemented materials in western mining areas. The concentration of rosin–alcohol solution has a measurable impact on key parameters, such as *σ_c_*, *E*, and *γ* in the weakly cemented similar material specimens. Furthermore, the creep characteristics of the specimens diminish progressively with an increase in the proportion of iron powder (I) and barite powder (B). The material was applied to a similar indoor model test simulating the weakly cemented material surrounding the auxiliary haulage roadway in Panel 20314 of the Gaojialiang Coal Mine, with speckle analysis employed for detailed examination. The experimental findings suggest that both the conventional mechanical properties and long-term creep characteristics of the material align with the required specifications, offering robust support for achieving optimal outcomes in the similar model test.

## 1. Introduction

In recent years, the focus of China’s coal mining has progressively shifted toward the coal-rich western regions. However, the soft rock strata found in these western mining areas differ significantly from those in the central and eastern regions of China. These rocks are characterized by macro-scale physical and mechanical properties, such as weak cementation, low strength, and susceptibility to water-induced slaking. Furthermore, they undergo creep deformation under relatively low stress levels, categorizing them as a special type of soft rock [1,2,3,4,5]. Such weakly cemented soft rock severely compromises the self-stability of roadway-surrounding rock in western mining areas, resulting in considerable long-term deformation and damage. This substantially impedes the safe and efficient development of coal resources in this region [6,7]. Consequently, a comprehensive understanding of the long-term creep behavior and failure mechanisms of weakly cemented soft rock, especially under shallow burial and low-stress environments, becomes of paramount engineering significance and profound scientific value for ensuring safe extraction in these areas.

Given the challenges associated with obtaining weakly cemented soft rock cores, including their inherent poor integrity and the vulnerability of their physical and mechanical properties to disturbance and deterioration, physical model testing, particularly excavation model testing using specially formulated similar materials, has emerged as an indispensable and effective methodology for investigating their deformation and failure characteristics [8,9,10,11,12].

A considerable body of research on soft rock and similar materials has been conducted by numerous scholars. Stück, H et al. [13] studied the physical properties of sandstone as a component in similar materials. Holtzman, R [14] investigated the micro-mechanical properties of weakly cemented rock, laying the groundwork for the composition of weakly cemented similar materials. Xu Ren et al. [15] developed an innovative soft rock material, utilizing fine iron powder, barite powder, and quartz sand as aggregates, with gypsum as a binder, and redispersible polymer powder as a modifier to simulate the stability issues of soft rock induced by excavation. Sun et al. [12] formulated coal measure rock-like materials based on materials like river sand, heavy calcium carbonate, cement, and gypsum, exploring the sensitivity of various factors. Qian Yongmei et al. [16] conducted tests on the mechanical properties of a new epoxy resin concrete, identifying specific mix ratios that significantly enhanced material performance while also refining the theoretical understanding of its tensile failure mechanism and structural design. Cui et al. [17] utilized the discrete element method to determine the optimal coarse-to-fine aggregate ratio for similar materials simulating weak surrounding rock. Li Yuanhai et al. [18], addressing the limitations of traditional physical model observations, proposed a method for preparing transparent rock masses, developing a transparent similar material suited for simulating soft rocks. Yang Xu et al. [19] determined appropriate, similar materials and mix ratios for soft rock by studying factors like the softening coefficient, tensile strength, and cohesion. Zhang Yanli et al. [20] conducted orthogonal experiments using river sand, coal powder, gypsum, and calcium carbonate as raw materials, determining key factors influencing the physical and mechanical properties of coal-like materials. Lin Haifei et al. [21], in developing “solid–gas” coupling-like materials, incorporated hydraulic oil as a regulator. They observed its favorable viscosity and observed that an increase in hydraulic oil content significantly reduced the material strength while elevating Poisson’s ratio, providing valuable guidance for the subsequent development of low-strength soft rock materials.

Regarding the creep characteristics of soft rock-like materials, Ma Zhenguo [22] selected styrene–butadiene latex as a binder to successfully formulate a soft rock-like material that could effectively simulate soft rock creep failure patterns. Wang Hanpeng et al. [23] used aggregates consisting of fine iron powder, barite powder, quartz sand, and quicklime to formulate an iron crystal sand cemented-like material, where the specimens exhibited good simulation of the instantaneous mechanical properties of medium- to low-strength rocks. Chu et al. [24,25,26], building upon previous research findings, developed a novel similar material for simulating soft rock creep. This material not only simulates the instantaneous elastoplastic properties of soft rock but also effectively mimics its creep characteristics. Furthermore, they employed a multiple linear regression method to determine the relationship between key factors and the mixture ratio of the similar material. This approach overcame the limitations of the trial-and-error method, which is often cumbersome and prone to randomness when determining the optimal mixture ratio. Consequently, the experimentally derived mixture ratios were significantly more accurate, and the process became significantly more efficient. Their advancements provided a robust foundation for the present study.

However, the research on materials similar to weakly cemented soft rock—characterized by substantial deformations, low strength, and significant creep behavior under low stress—remains insufficient. To better characterize the creep properties of weakly cemented soft rock and overcome the challenges of coring and preserving such cores, this paper first determines the target mix ratio based on existing multiple linear regression equations for soft rock creep and similar materials [25]. Secondly, the influence of various components on the conventional mechanical parameters and creep characteristics of specimens is investigated through orthogonal tests (three factors, three levels) and proportional tests to determine a suitable mix ratio for weakly cemented soft rock. Building on this, using the auxiliary haulage roadway of the 20314 working face in the Gaojialiang Coal Mine as a case study, a two-dimensional plane model test is conducted employing the identified weakly cemented soft rock-like material. The digital speckle correlation method (DSCM) is utilized for analysis to study the creep failure mechanism of weakly cemented surrounding rock roadways induced by shallow burial and low stress, thereby providing both material assurance and a theoretical foundation for subsequent research on weakly cemented soft rock.

## 2. Research Background

### 2.1. Project Overview

**Figure 1:**
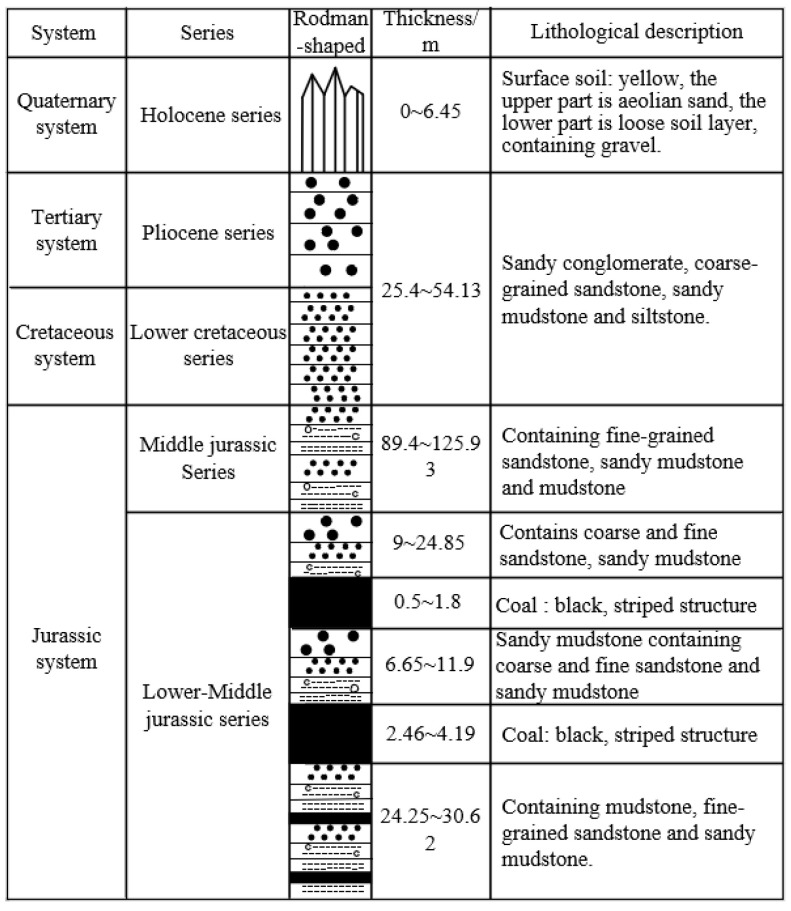
Geological comprehensive histogram of the Gaojialiang Coal Mine.

The primary coal seams mined at the Gaojialiang Coal Mine include the 2-2 upper, 2-2 middle, 3-1, 4-2 middle, 5-1, and 6-2 middle seams. These coal seams are characterized by stable stratigraphic positions and simple structures (Figure 1).

The 20314 working face mines the 2-2 middle coal seam, located in the 203 panel. The ground elevation at the working face ranges from 1445.8 m to 1499.0 m, while the elevation of the coal seam floor varies between 1281.4 m and 1297.9 m. The working face spans a strike length of 300.5 m and a dip length of 1459.2 m, with recoverable reserves amounting to 2.423 million tons. The coal seam thickness ranges from 3.20 m to 4.70 m, averaging 4.06 m, with a dip angle of 0–5°. The design employs full-seam mining in a single pass.

The 20314 adequately mechanized working face is arranged along the strike of the coal seam, while the working face roadways are oriented along the dip direction of the 2-2 middle coal seam.

As depicted in Figure 1, the immediate roof and floor of the coal seam consist of weakly cemented sandy mudstone. Due to the short diagenetic time, low cementation degree, loose rock mass structure, and well-developed fractures in the surrounding rock, the strength of the roof and floor strata is lower than that of the coal seam itself.

### 2.2. Deformation and Failure Characteristics of Roadway

**Figure 2:**
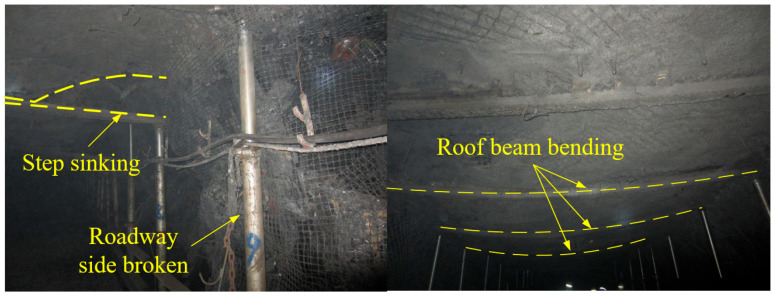
On-site failure characteristics.

The haulage roadway of 20314 fully mechanized mining face exhibits various forms of structural distress, including floor fractures, roof beam bending, stepped roof subsidence, severe floor heave, and failure of the support system (as revealed in Figure 2). These substantial roadway deformations impede its normal functionality.

This is predominantly attributable to the properties of the shallow, buried, weakly cemented strata, which exhibit a low diagenetic degree, poor cementation, and creep behavior under low stress levels, compounded by the influence of the overlying goaf, which results in significant mining disturbance effects. Subsequent to the completion of mining in the upper working face, the residual coal pillars above generate concentrated stress on the haulage roadway, thereby inducing creep deformation and subsequent failure of the weakly cemented medium in the roadway roof.

## 3. Development of Weakly Cemented Similar Materials

### 3.1. Similarity Criteria and Model for Weakly Cemented Materials

Given the intrinsic challenges associated with studying the deformation and failure conditions of weakly cemented strata roadways in situ, coupled with the difficulties in coring and preserving weakly cemented medium samples from western regions, the method of similar material simulation has been employed to investigate the creep deformation and failure characteristics of weakly cemented mediums. To accurately simulate these characteristics, it is imperative to determine an appropriate model similarity scale and to develop similar materials that sufficiently replicate the creep properties of weakly cemented medium.

When considering the creep effects of weakly cemented medium, both conventional physical–mechanical parameters and time-dependent creep mechanical parameters must be considered. The conventional physical–mechanical parameters include the geometric dimension *L*, unit weight *γ*, stress *σ*, strain *ε*, elastic modulus *E*, Poisson’s ratio *μ*, cohesion *C*, internal friction angle *φ*, and uniaxial compressive strength *σ_c_*. In accordance with similarity theory, the ratio of physical quantities with the same dimension between the prototype material (*P*) and the similar model (*M*) is referred to as the similarity scale, denoted by *C_X_* (where *X* represents each parameter).

By considering the equilibrium equations, geometric equations, physical equations, stress boundary conditions, and displacement boundary conditions of the prototype material and the similar model, the following similarity associations for the similar model test can be derived [27]:

(1) $$\left\{\begin{array}{l} C_{\sigma }=C_{\gamma }C_{L}\\ C_{\delta }=C_{\varepsilon }C_{L}\\ C_{\sigma }=C_{\varepsilon }C_{E}\\ C_{\varepsilon }=C_{f}=C_{\varphi }=C_{\mu }=1\\ C_{\sigma }=C_{E}=C_{c}=C_{\sigma_{c}} \end{array}\right.$$

**Figure 3:**
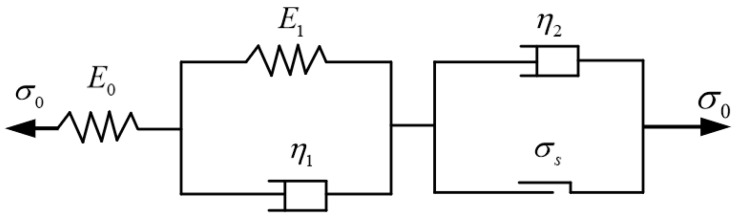
Ishihara rheological model.

The meaning of each symbol is provided in Nomenclature. The creep mechanical parameters of the weakly cemented medium are determined by its creep constitutive correlation. Both the Nishihara model (as revealed in Figure 3) and the Burgers model are widely recognized theoretical models used to describe the viscoelastic–plastic behavior of soft medium, and both are utilized to simulate the rheological characteristics of soft medium. Nevertheless, Tao et al. [28] put forth a standpoint that the Nishihara model is more suitable than the Burgers model for describing the creep properties of soft medium. Consequently, the parameters of the Nishihara model are adopted as the creep similarity parameters between the weakly cemented similar material and the prototype material. Given the experimental operability, the corresponding creep parameters are determined through uniaxial creep compression tests.

The one-dimensional Nishihara creep equation is

(2) $$\varepsilon (t)=\left\{\begin{array}{c} \frac{\sigma_{0}}{E_{0}}+\frac{\sigma_{0}}{E_{1}}[1-exp(-\frac{E_{1}}{\eta_{1}}t)],\sigma_{0}<\sigma_{s}\\ \frac{\sigma_{0}}{E_{0}}+\frac{\sigma_{0}}{E_{1}}[1-exp(-\frac{E_{1}}{\eta_{1}}t)]+\frac{\left(\sigma_{0}-\sigma_{s}\right)}{\eta_{2}}t,\sigma_{0}>\sigma_{s} \end{array}\right.$$

The creep rate of the Nishihara model is

(3) $$\dot{\varepsilon }(t)=\left\{\begin{array}{c} \frac{\sigma_{0}}{\eta_{1}}e^{-\frac{E_{1}}{\eta_{1}}},\sigma_{0}<\sigma_{s}\\ \frac{\sigma_{0}}{\eta_{1}}e^{-\frac{E_{1}}{\eta_{1}}}+\frac{\sigma_{0}-\sigma_{s}}{\eta_{2}},\sigma_{0}>\sigma_{s} \end{array}\right.$$

where: *ε* refers to the strain; *σ*_0_ symbolizes the creep stress; *E*_0_ represents the instantaneous elastic modulus; *E*_1_ denotes the viscoelastic modulus; *η*_1_ and *η*_2_ represent the viscosity coefficients; *σ_s_* symbolizes the long-term strength; $\dot{\varepsilon }$ denotes the strain rate.

Scholars have [29] highlighted that creep-induced deformation and failure are time-dependent (correlated with time *t*), which means that the conventional time similarity association $C_{t}=\sqrt{C_{L}}$ cannot be applied. Assuming the similarity ratios of viscosity coefficients *η*_1_ and *η*_2_ as *C_η_*, and the strain rate similarity ratio as $C_{\dot{\varepsilon }}$, it can be deduced from Equation (1) that the similarity ratios of creep stress *σ*_0_, viscoelastic moduli *E*_1_ and *E*_2_ should be $C_{\sigma }$, while the strain *ε* similarity ratio $C_{\varepsilon }=1$. For this reason, the parameters for the prototype materials and the similar materials should satisfy the following connections [24]:

(4) $$\left\{\begin{array}{c} \sigma_{0,p}=C_{\sigma }\sigma_{0,m},t_{p}=C_{t}t_{m}\\ E_{0,p}=C_{\sigma }E_{0,m},E_{1,p}=C_{\sigma }E_{1,m},E_{2,p}=C_{\sigma }E_{2,m}\\ \eta_{1,p}=C_{\eta }\eta_{1,m},\eta_{2,p}=C_{\eta }\eta_{2,m}\\ \varepsilon_{p}=\varepsilon_{m},{\dot{\varepsilon }}_{p}=C_{\dot{\varepsilon }}{\dot{\varepsilon }}_{m} \end{array}\right.$$

In the equation, subscripts “*p*” and “*m*” represent the prototype materials and similar material, respectively. The one-dimensional creep equation for both the prototype materials and similar materials is calculated as. follows:

(5) $$\varepsilon_{t}=\left\{\begin{array}{c} \frac{\sigma_{0}}{E_{0}}+\frac{\sigma_{0}}{E_{1}}[1-exp(-\frac{E_{1}}{\eta_{1}}t)],\sigma_{0}<\sigma_{s}\\ \frac{\sigma_{0}}{E_{0}}+\frac{\sigma_{0}}{E_{1}}[1-exp(-\frac{E_{1}}{\eta_{1}}t)]+\frac{\sigma_{0}-\sigma_{s}}{\eta_{2}}t,\sigma_{0}>\sigma_{s} \end{array}\right.$$

Substituting Equation (4) into Equation (5), and simplifying, yields

(6) $$\varepsilon_{p}(t_{p})=\left\{\begin{array}{c} \frac{C_{\sigma }\sigma_{0,m}}{C_{\sigma }E_{0,m}}+\frac{C_{\sigma }\sigma_{0,m}}{C_{\sigma }E_{1,m}}[1-exp(-\frac{C_{\sigma }E_{1,m}}{C_{\eta }\eta_{1,m}}C_{t}t_{m})],\sigma_{0,m}<\sigma_{s,m}\\ \frac{C_{\sigma }\sigma_{0,m}}{C_{\sigma }E_{0,m}}+\frac{C_{\sigma }\sigma_{0,m}}{C_{\sigma }E_{1,m}}[1-exp(-\frac{C_{\sigma }E_{1,m}}{C_{\eta }\eta_{1,m}}C_{t}t_{m})]+\frac{C_{\sigma }(\sigma_{0,m}-\sigma_{s,m})}{C_{\eta }\eta_{2,m}}C_{t}t_{m},\sigma_{0,m}>\sigma_{s,m} \end{array}\right.$$

From the above, it follows that $C_{\varepsilon }=1$, meaning that for any time *t*, the strain of the prototype materials equals that of the similar material, i.e., $\varepsilon_{p}(t_{p})=\varepsilon_{m}(t_{m})$. Therefore, the similarity ratios *C_η_*, *C_σ_*, and *C_t_* can be derived as

(7) $$C_{\eta }=C_{t}C_{\sigma }$$

By substituting Equation (4) into (3) and considering Equation (5), we obtain

(8) $$C_{\dot{\varepsilon }}=1/C_{t}$$

On that account, when *C_σ_* is constant, larger *C_t_* values lead to greater *C_η_*_1_ and *C_η_*_2_ values. This indicates that the viscosity coefficients of the similar material decrease while its creep rate increases. Rooted in this principle, materials with higher creep rates must be selected to simulate the long-term creep effects of weakly cemented medium within a short timeframe.

### 3.2. Material Selection and Similarity Ratio Determination

Chu et al. developed a similar material that effectively simulates the rheology of soft rock using iron ore powder, heavy stone powder and quartz sand as aggregate, with rosin alcohol solution as the cementing agent and hydraulic oil as the adhesive [24,25,26]. Therefore, the similar materials for this test utilized quartz sand, iron ore powder (300 mesh) and barite powder as aggregates. For the binder, a rosin–alcohol solution was selected, with 99% pure industrial alcohol used as the solvent to dissolve the rosin. Subsequent to alcohol volatilization, the rosin formed cementation force. Grade 6 industrial hydraulic oil was chosen as the viscous agent.

Grounded in parameters of weakly cemented medium and the connection between various factors and mix proportion of similar materials, this paper selected the unit weight γ, elastic modulus *E*, internal friction angle *φ*, and viscosity coefficient *η*_1_ as the main control parameters of similar materials [25]. Let the rosin concentration *n* be *X*_1_, the mass ratio of barite powder to iron powder (*IB/IBS*) be *X*_2_, the mass ratio of iron powder to the sum of barite and iron powder (*I/IB*) be *X*_3_, and the hydraulic oil content *ω* be *X*_4_. Through multiple linear regression analysis, the material mix proportion can be determined. The regression equation is as follows [25]:

(9) $$\left.\begin{array}{l} \gamma =-2.64X_{1}+5.6X_{2}+4.4X_{3}+3X_{4}+20.7\\ E=1175.5X_{1}+142.9X_{2}+103.2X_{3}-5269.3X_{4}+84.8\\ \varphi =-8.6X_{1}-20.6X_{2}+0.63X_{3}-58X_{4}+56.2\\ \eta_{1}=874.7X_{1}-80.8X_{2}+98.5X_{3}-3851.3X_{4}+171.4 \end{array}\right\}$$

Formula (9) is inversely solved to obtain the material ratio expression:

(10) $$\left.\begin{array}{l} X_{1}=-0.026\gamma -0.0026E-0.0411\varphi +0.0041\eta_{1}+2.357\\ X_{2}=0.021\gamma +0.0031E-0.0049\varphi -0.0042\eta_{1}+0.293\\ X_{3}=0.1868\gamma -0.005E-0.012\varphi +0.0071\eta_{1}-3.99\\ X_{4}=-0.0016\gamma -0.0008E-0.0095\varphi +0.0009\eta_{1}+0.472 \end{array}\right\}$$

## 4. Test Scheme and Process

### 4.1. Orthogonal Test Design

In the orthogonal test design, the mass of the rosin–alcohol solution was fixed at 6% of the total aggregate mass. The test primarily focused on three key control factors: first, the ratio of iron powder mass to the total mass of barite powder and iron powder; second, the ratio of the total mass of barite powder plus iron powder to the aggregate mass; and third, the concentration of the rosin–alcohol solution. For these factors, three dissimilar levels were established for the experimental research.

Notably, the level ranges for aggregates and binders were determined with reference to Chu, Zhang et al. [24,30]. The hydraulic oil content (*ω*) was determined based on its influence on similar materials, and the results obtained from multiple linear regression equation calculations. The final experimental design is presented in Table 1, while the mix proportion scheme is outlined in Table 2. Let the rosin concentration be *n*, the mass ratio of barite powder to iron powder be (*IB/IBS*), the mass ratio of iron powder to the sum of barite and iron powder be (*I/IB*), and the hydraulic oil content be *ω*.

### 4.2. Sample Preparation and Testing Process

#### 4.2.1. Sample Preparation and Testing Procedure

**Figure 4:**
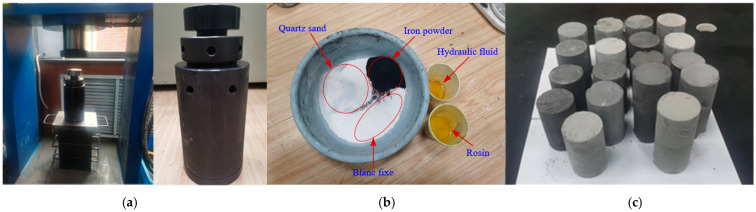
Mold and preparation process. (**a**) Special structured mold. (**b**) Similar material display diagram before mixing. (**c**) Formed specimens.

In accordance with the ratio outlined in Table 2, standard specimens with a diameter of 50 mm and a height of 100 mm were made in strict compliance with the recommended standards of the International Society of Rock Mechanics (ISRM). The specific test steps are as follows:

(1) Material preparation: Raw materials were prepared in line with the proportions specified in Table 2.
(2) Mold preparation: Pre-compression is essentially ascribable to the special characteristics of the materials adopted in this test and the relatively low strength of the prepared samples. Conventional soil sample molds would have resulted in demolding difficulties, rendering pre-compression unfeasible. Consequently, specially designed structured molds were employed, as depicted in Figure 4a.
(3) Quartz sand, barite powder, iron powder, hydraulic oil, and rosin–alcohol solution were individually weighted. Afterward, the measured aggregates were combined in a container for mixing. After thorough mixing, the rosin–alcohol solution was added sequentially. Once the solution had been fully absorbed, hydraulic oil was incorporated, and the mixing process continued. After completion, the mixture was set aside for later use, and the mold walls were lubricated with silicone oil, as displayed in Figure 4b.
(4) The mixed material was loaded into the newly designed mold cavity, and manual compaction was carried out. The compacted material volume was controlled to approximately 120% of that of the specimen.
(5) Pressing: The mold plunger was installed, and pressure was applied using an electro-hydraulic press, allowing the formation of standard specimens for rock mechanics testing.
(6) Demolding: The mold plunger was installed, and pressure was applied using an electro-hydraulic press, facilitating the formation of standard rock mechanics test specimens.
(7) As indicated by *Z* in Table 2, a total of 9 test groups were conducted. Each group consisted of 4 specimens: 3 specimens for uniaxial compression tests and 1 specimen for uniaxial creep tests.
(8) The specimens were numbered and cured under room-temperature and dry conditions for 10 days, allowing the alcohol to fully evaporate. The cured specimens are illustrated in Figure 4c.

**Figure 5:**
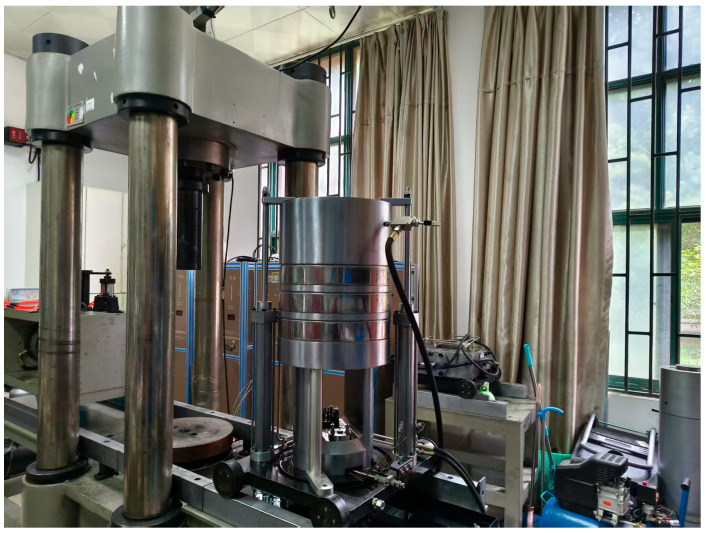
Basic mechanical test system.

Basic physical and mechanical tests were conducted to investigate the cured specimens. The physical tests included density and dimension measurements, where the density and dimensions of the specimens were assessed to obtain the average density *γ* for each mix ratio. The mechanical tests comprised uniaxial compression tests and uniaxial creep tests, which were performed using the RLJW-2000 mechanical test system (Produced in Qingdao, China). The uniaxial compression test was used to measure the Poisson’s ratio *μ*, elastic modulus *E*, and uniaxial compressive strength *σ_c_* of the specimens, while the uniaxial creep test captured the creep deformation characteristics of the specimens. The testing system is demonstrated in Figure 5.

The steps for the uniaxial compression test were as follows:

(1) The prepared similar material specimen was positioned at the center of the pressure head of the testing machine, and a circumferential extensometer and axial strain gauge was attached to it.
(2) Loading began at a displacement-controlled rate of 0.05 mm/s until specimen failure occurred.

The steps for the uniaxial creep test were as follows:

(1) The prepared similar material specimen was placed at the center of the testing machine’s pressure head, and an axial strain gauge was attached to it.
(2) Loading began at a stress-controlled rate of 0.001 MPa/s until the specimen failed.

#### 4.2.2. Physical Test Results and Analysis

(a) Conventional Physical Test Results and Analysis

**Figure 6:**
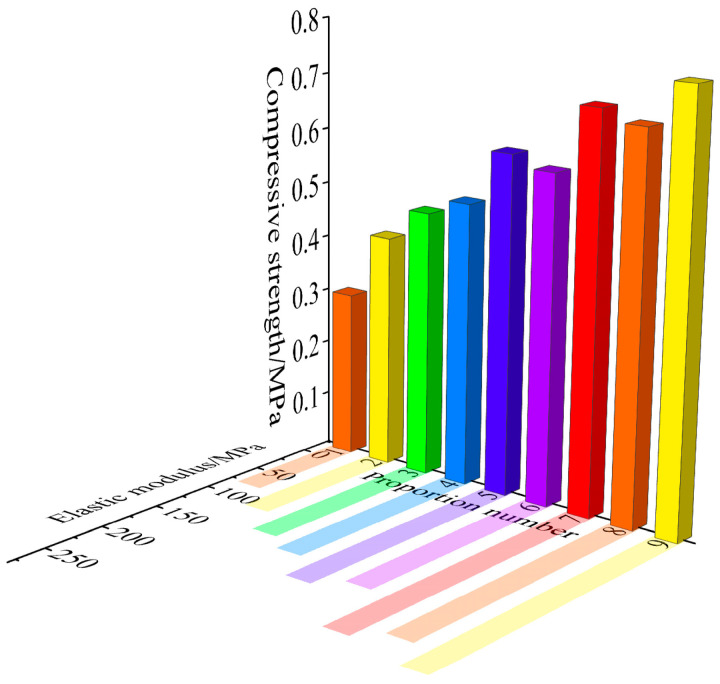
Variation in elastic modulus and compressive strength.

As revealed in Table 3 and Figure 6, the conventional physical and mechanical parameters for each mix ratio were obtained from the uniaxial compression tests. A comprehensive analysis of the data from each mix ratio revealed the following: For this weakly cemented medium simulant material, the bulk density (*γ*) of the mix ratios 1 to 9 ranged from 24.32 to 26.91 kN/m^3^, the elastic modulus (*E*) ranged from 79.4 to 241.05 MPa, the uniaxial compressive strength ranged from 0.30 to 0.77 MPa, and the Poisson’s ratio ranged from 0.21 to 0.27. The variation in bulk density for this simulant material was relatively modest, aligning closely with the typical bulk density range observed in soft rocks.

**Figure 7:**
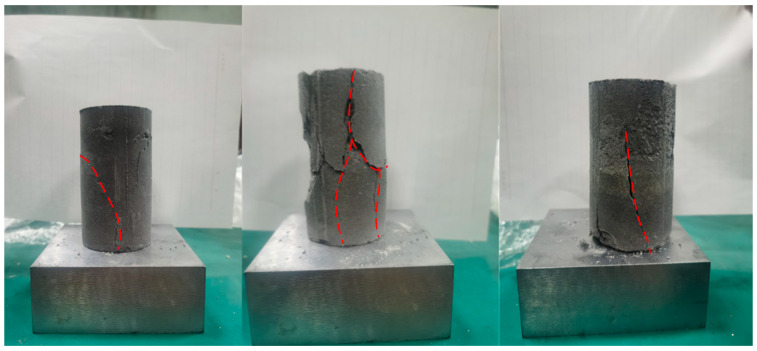
Macroscopic failure state of specimen under uniaxial compression test.

Figure 7 depicts the rupture pattern of certain test specimens. As illustrated, the specimens exhibited vertical tensile failure. Under loading, transverse tensile stresses were induced, accompanied by a noticeable Poisson’s effect. Crack propagation in the specimens progressed gradually along the axial direction, eventually culminating in structural failure. This process was accompanied by the formation of minor secondary cracks adjacent to the primary penetrating cracks.

In accordance with the standard [31], the uniaxial compressive strength of engineering soft medium typically falls below 15 MPa. Rooted in Equation (1) and the previously determined ranges for the density similarity ratio and geometric similarity ratio in similar material simulation tests [25], it can be inferred that the stress similarity ratio *C_σ_* should typically exceed 15. Consequently, the uniaxial compressive strength of weakly cemented similar materials should generally be below 1 MPa.

The data presented in Table 3 and Figure 6 confirm that the weakly cemented similar materials prepared in this study satisfactorily conform to the required strength range and other mechanical parameter requirements.

**Figure 8:**
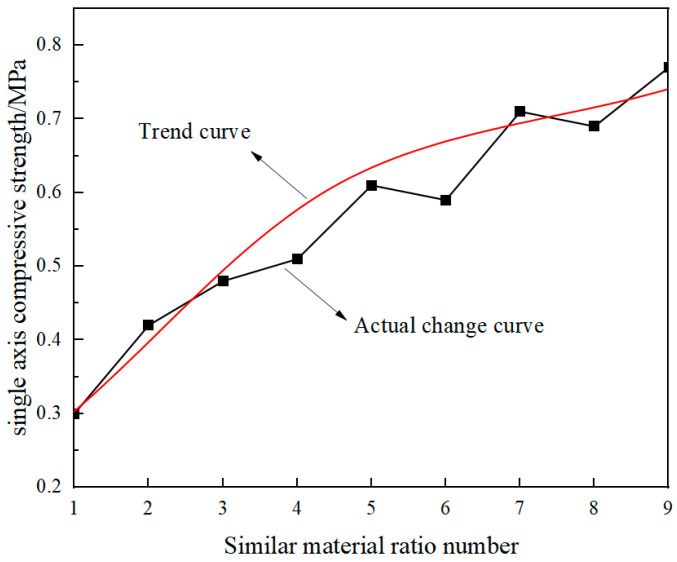
The variation law of uniaxial compressive strength of specimens with diverse ratios.

The results of the uniaxial compression tests on similar materials are depicted in Figure 8. As the concentration of rosin alcohol increased, the overall compressive strength of the specimens gradually rose, illustrating that the rosin–alcohol solution had a notable impact on enhancing the compressive strength of the specimens. This finding aligns with the conclusions drawn by Dong et al. [32], thereby validating the reliability of the similar material tests.

(b) Results and Analysis of Uniaxial Creep Tests

Table 4 presents the mechanical parameters of weakly cemented soft materials. Based on the previously determined similarity scale, the mechanical parameters of the similar materials were calculated for the target specimens, with the specific results shown in Table 5. A comprehensive comparison demonstrates that the proportions of Mix No. 4 and Mix No. 5 basically satisfy the strength and other parameter requirements for the weakly cemented similar materials necessary for this study. As a consequence, uniaxial creep tests were conducted on specimens prepared with the proportions of Mix No. 4 and Mix No. 5.

**Figure 9:**
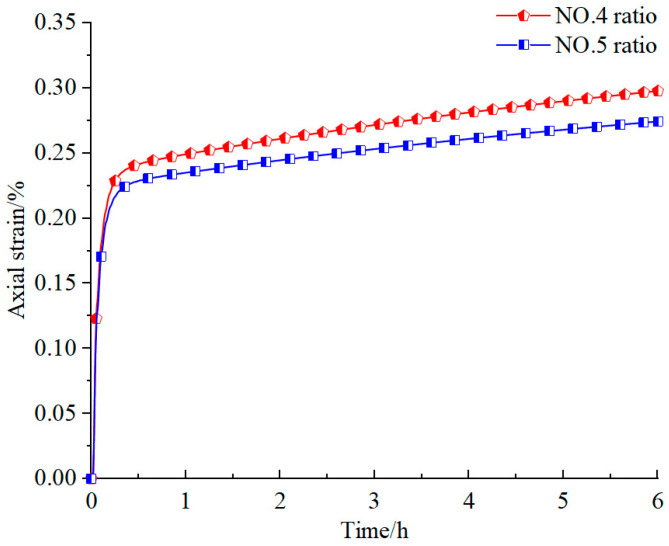
Creep test curves of similar material specimens with dissimilar proportions.

As demonstrated in Figure 9, the creep test curves of specimens with varying material ratios exhibited distinct characteristics. The axial strains of specimens with ratios No. 4 and No. 5 show a marked increase during the initial stage, followed by a gradual deceleration in the strain rate over time. This phenomenon illustrates that both specimens enter the decay creep stage at this point. After the strain rates stabilize at 0.23% and 0.22%, separately, the rate of strain growth strikingly slows down, eventually transitioning into a steady and uniform increase. This demonstrates that the weakly cemented similar material specimens, prepared according to ratio schemes No. 4 and No. 5, demonstrate favorable creep characteristics, with their creep curves closely resembling the shape of real creep curves, meeting the experimental requirements. Simultaneously, as the proportion of iron powder (I) and barite powder (B) increases, the creep characteristics of the specimens gradually diminish.

## 5. Model Test

### 5.1. Model Design

Drawing from the auxiliary transportation roadway of the 20314 working face at the Gaojialiang Coal Mine, Ordos City, Inner Mongolia Autonomous Region, a plane model test was conducted to investigate the creep failure mechanism of weakly cemented stratum medium roadways induced by shallow burial and low stress. Given that roadway excavation induces stress redistribution, and the stress essentially reverts to its original state beyond five times the chamber radius, an influence range of eight times the chamber radius was chosen to streamline calculations and reduce errors.

**Figure 10:**
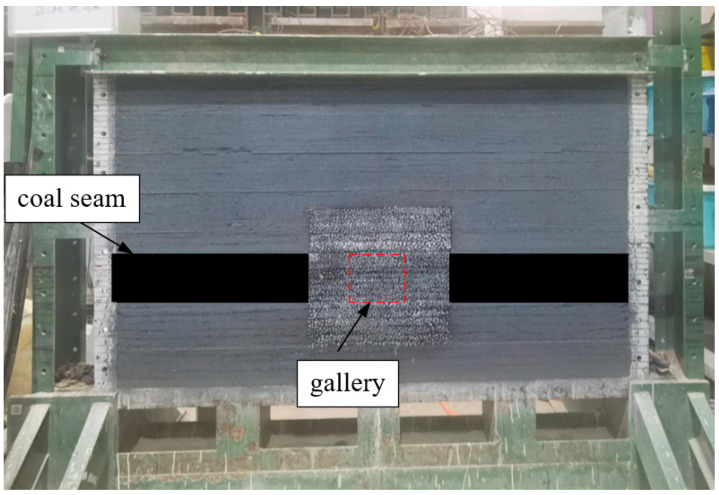
Simulator stand.

The auxiliary transportation roadway of the 21304 working face has a width of 5 m and a height of 3.5 m, which led to the determination of an influence range of 40 m × 28 m. The model test platform employed in this experiment has dimensions of 2.0 m × 0.22 m × 1.8 m (as revealed in Figure 10).

In this model test, the fundamental similarity ratios of the materials were as follows:

(11) $$\left.\begin{array}{r} C_{L}=\frac{\delta_{P}}{\delta_{m}}=\frac{40m}{2m}=20\\ C_{\sigma }=C_{\gamma }C_{L}=20\times 0.83\approx 17\\ C_{t}=4\\ C_{\eta }=C_{t}C_{\sigma }=4\times 17=68 \end{array}\right\}$$

In the equation, *C_L_* denotes the geometric similarity ratio of the model, *C_t_* symbolizes the time similarity ratio, and *C_η_* refers to the viscosity coefficient similarity ratio. The formation model proportionately selected schemes No. 4, No. 5, and No. 6 to simulate weakly cemented mudstone, weakly cemented sandy mudstone, and coal (the coal seam refers to Xu [33]), as presented in Table 6.

### 5.2. Test Process and Monitoring

This experiment was conducted on a two-dimensional test platform, serving as the model stand, with fixed boundaries on the front, back, left, right, and lower sides. To minimize boundary effects, the surface was smoothed prior to laying the strata. Throughout the testing process, the strata similarity materials were prepared in accordance with the ratios in Table 6, and the strata were subsequently applied in layers. Mica powder was uniformly distributed between each layer to act as a weak interlayer, ensuring consistency and stratum homogeneity. After the model was completed and dried, an equivalent concentrated load of 0.132 MPa was applied to the top of the model to simulate the concentrated stress exerted by the residual coal pillars above. Simultaneously, the excavation of the 2-2 middle coal seam roadway commenced, accompanied by continuous monitoring.

**Figure 11:**
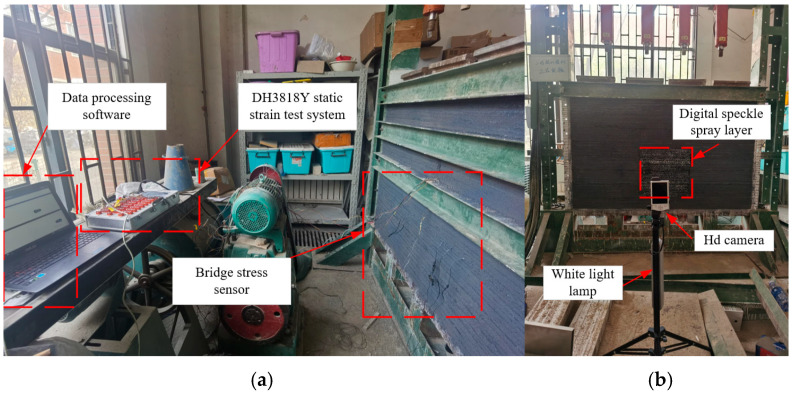
Monitoring system. (**a**) DH3818Y static strain test system. (**b**) Speckle monitoring.

**Figure 12:**
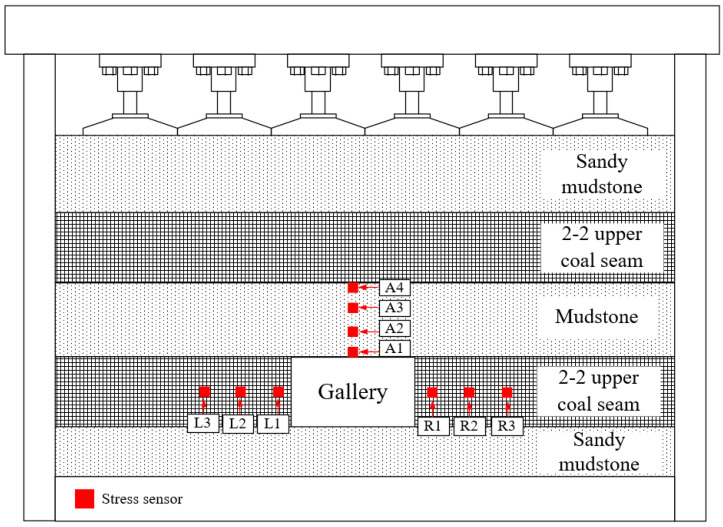
Stress sensor measuring point arrangement.

As shown in Figure 11 and Figure 12, the comprehensive monitoring of the entire model encompassed stress monitoring, roadway deformation, and macroscopic failure monitoring.

The stress monitoring system utilized the DH3818Y static strain gauge (Produced in Qingdao, China), which operated on the principle of static stress testing to track stress variations via bridge-type stress sensors embedded in the model. A total of 10 measurement points were strategically positioned in the roof and sidewalls of the roadway.

As illustrated in Figure 11b, the digital speckle testing system comprised a high-definition camera, supplementary lighting, speckle spray paint, and speckle analysis software(Ncorr v1.2.2). Black primer and white speckle dots were sprayed around the roadway, with the camera set to automatically capture images every 5 min. Following roadway excavation, the weakly cemented soft medium underwent deformation under stress, causing displacement of the speckle dots on the roadway surface. The captured images were subsequently uploaded to the analysis software, enabling the determination of the deformation characteristics and macroscopic fracture behavior of the roadway.

### 5.3. Analysis of Physical Model Test Results

(1) Evolution Law of Weakly Cemented Soft Medium

**Figure 13:**
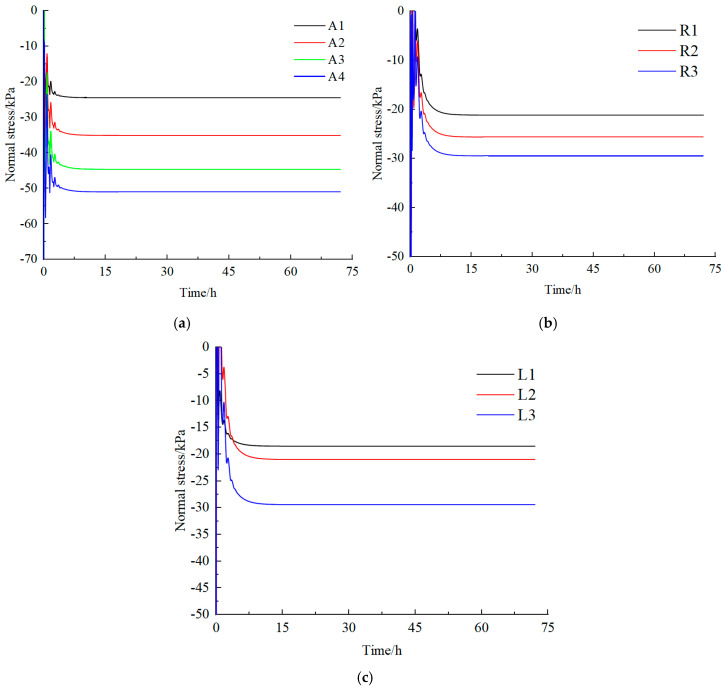
Vertical stress–time curve of measuring point. (**a**) Roof station. (**b**) Measuring points on left side of roadway. (**c**) Measuring points on the right side of roadway.

Figure 13 illustrates the stress evolution curves of the measurement points over time. During the initial stage, the model experienced a rapid stress release, causing a redistribution of stress that resulted in significant fluctuations in the stress curves of the roof and both sidewalls. Overall, the stress at the measurement points in the roadway gradually increased over time, with the stress diminishing as the distance from the roadway increased.

The stress in the roof of the model was more pronounced than that in the two sides. This phenomenon can be attributed to the concentration of load above the roof of the roadway. After 10 h of excavation, the trend of stress increase began to decelerate and eventually stabilized. At this point, the stress value of the roof ranged between −24.49 and −50.99 kPa, while the stress value of the sides ranged between −18.5 and 29.46 kPa. The ratio of side stress to roof stress was between 0.36 and 0.83, demonstrating that stress was mainly concentrated in the roof area. By comparing the stress conditions of the roof and side measurement points over time, it is evident that the roadway deformation exhibited significant creep characteristics.

(2) Surface Deformation and Failure Patterns of Weakly Cemented Soft Medium

**Figure 14:**
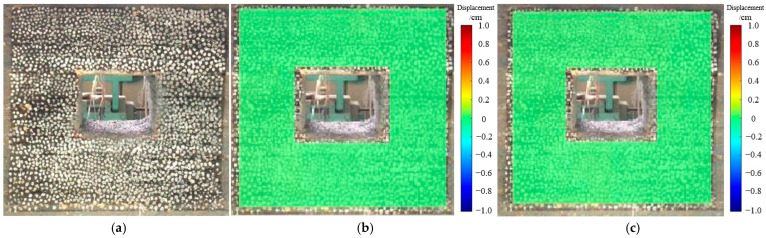
Deformation and failure of roadway after initial excavation. (**a**) Characteristics of roadway failure. (**b**) Horizontal displacement situation. (**c**) Vertical displacement situation.

**Figure 15:**
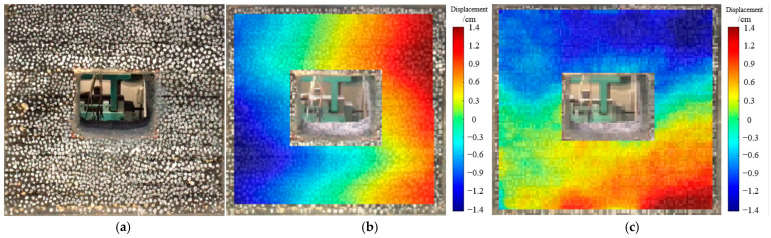
Deformation and failure of roadway after excavation for 1 day. (**a**) Characteristics of roadway failure. (**b**) Horizontal displacement situation. (**c**) Vertical displacement situation.

**Figure 16:**
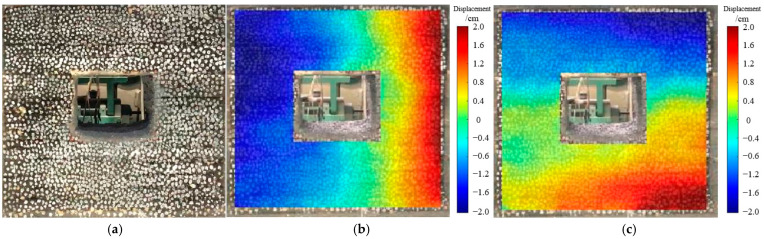
Deformation and failure of roadway after excavation for 3 days. (**a**) Characteristics of roadway failure. (**b**) Horizontal displacement situation. (**c**) Vertical displacement situation.

In accordance with the experimental design, the roadway was excavated, and the digital speckle method was employed to accurately capture the deformation of the roadway sides and roof. The experimental results are depicted in Figure 14, Figure 15 and Figure 16.

**Figure 17:**
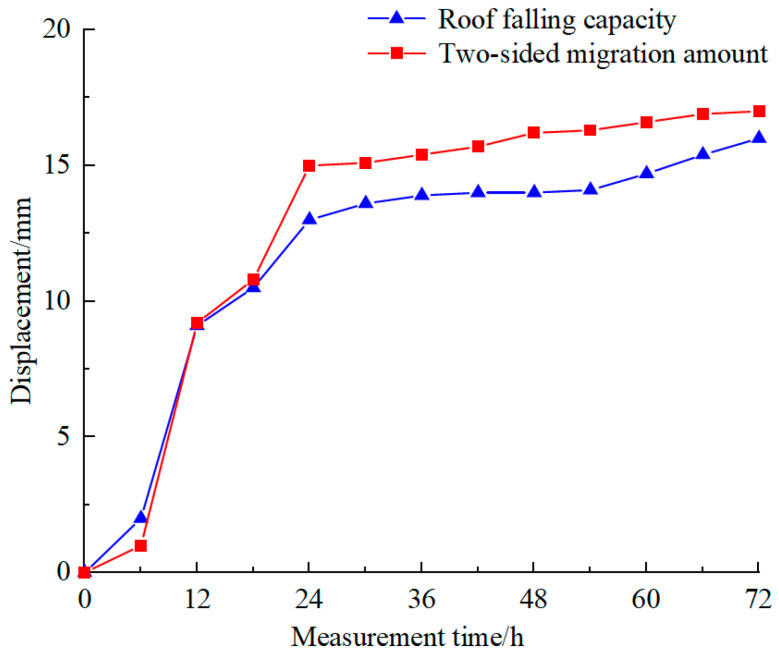
Variation law of surface displacement of roadway.

As shown in Figure 14, during the initial excavation, the stress above the roadway was rapidly released, resulting in deformation. Nonetheless, the overall stability of the roadway was maintained. As shown in Figure 17, The displacement of the roof and sidewalls was relatively small, with the maximum roof subsidence approximately 2 mm and the maximum sidewall convergence about 1 mm, both exhibiting a symmetrical distribution.

As revealed in Figure 15, one day after excavation, the vertical deformation of the roadway was predominantly characterized by roof subsidence, which reached approximately 13 mm. The deformation pattern of the sidewalls shifted from a symmetrical to an asymmetrical distribution, with the left sidewall exhibiting greater deformation than the right sidewall.

As depicted in Figure 16, the movement of the roadway roof and floor continued to show roof subsidence, with a maximum subsidence of 16 mm. The deformation of the side walls was principally concentrated near the center, with a maximum convergence of 17 mm.

As depicted in Figure 17, following the excavation of the roadway, creep deformation progressively occurred under concentrated loads. During the initial excavation stage, creep deformation was induced by concentrated loads. Ascribable to the relatively high overall strength, the deformation of the roof and sidewalls remained minimal at this stage, with the sidewall deformation showing a symmetrical distribution. Subsequently, the roadway began to exhibit clear creep characteristics under the action of concentrated loads. Throughout this phase, the rate of deformation accelerated, with the convergence of the sidewalls and subsidence of the roof increasing strikingly. Nonetheless, as the excavation time progressed, the stress of the roadway gradually stabilized, and the deformation transitioned from a sharp increase to a steady state. At this stage, the convergence of the sidewalls exceeded the subsidence of the roof, and the sidewall deformation shifted from a symmetrical to an asymmetrical distribution.

## 6. Conclusions

This study, building upon the existing multivariate linear regression equation for the new soft medium, not only determined the target specimen proportion range but also identified the elastoplastic and creep characteristics of the novel material through orthogonal proportion tests. Additionally, a two-dimensional model test was conducted to evaluate the stability of the auxiliary transportation roadway in the 20314 working face of the Gaojialiang Coal Mine, considering long-term stratum load as the background. The test revealed the deformation and stress variation patterns in weakly cemented formation medium roadways. The main research findings are as follows:

(1) A similar material to simulate weakly cemented soft rock was successfully developed. The similar materials exhibited low strength and pronounced creep properties, making them particularly suitable for simulating weakly cemented medium in western mining areas. This methodology offers a brand new material selection option for conducting model tests on weakly cemented formation medium in these regions.
(2) The results from the orthogonal tests reveal that the concentration of rosin–alcohol solution significantly influenced critical parameters, such as the *σ_c_*, *E*, and *γ* of the weakly cemented similar material specimens. Creep tests on specimens 4 and 5 demonstrated that as the proportion of iron powder (I) and barite powder (B) increased, the creep characteristics of the specimens progressively weakened.
(3) When this similar material was employed in the similarity model test to evaluate the surrounding rock stability of the auxiliary transportation roadway in Panel 20314 of the Gaojialiang Coal Mine under long-term stratum load, the test results indicate that approximately 10 h after excavation, the stress measured at the roof and sidewall monitoring points gradually slowed and eventually stabilized. As the excavation continued, the surrounding rock deformation transitioned from a sharp increase to gradual stabilization, shifting from a symmetrical to an asymmetrical distribution. This indicates that under the combined influence of concentrated load and creep load, the surrounding rock of the roadway underwent creep deformation. Both the conventional mechanical properties and long-term creep characteristics of this material satisfied the test requirements, effectively simulating the long-term creep behavior of weakly cemented soft rock in shallow buried strata. This provided reliable material support for achieving favorable test results.

## Figures and Tables

**Table 1:** Orthogonal test levels of similar materials.

Level	*n*/%	*IB/IBS*	*I/IB*	*ω*/%
1	7.5	0.6	0.05	3.5
2	12.5	0.7	0.2	3.5
3	17.5	0.8	0.35	3.5

**Table 2:** Proportioning scheme of similar materials.

Proportion Number	*n*/%	*IB/IBS*	*I/IB*	*ω*/%
1	7.5	0.6	0.05	3.5
2	7.5	0.7	0.20	3.5
3	7.5	0.8	0.35	3.5
4	12.5	0.6	0.20	3.5
5	12.5	0.7	0.35	3.5
6	12.5	0.8	0.05	3.5
7	17.5	0.6	0.35	3.5
8	17.5	0.7	0.05	3.5
9	17.5	0.8	0.20	3.5

**Table 3:** Results of conventional physical and mechanical parameters.

Proportion Number	Volumetric Weight γ/(kN·m^−3^)	Elastic Modulus *E/*MPa	Compressive Strength *σ_c_/*MPa	Poisson Ratio *μ*
1	24.32	79.4	0.30	0.27
2	25.25	109.2	0.42	0.26
3	26.91	138.97	0.48	0.25
4	24.17	153.69	0.51	0.23
5	25.64	183.46	0.61	0.23
6	24.98	166.79	0.59	0.24
7	25.24	227.95	0.71	0.21
8	24.33	211.28	0.69	0.22
9	25.57	241.05	0.77	0.22

**Table 4:** Mechanical parameters of weakly cemented materials.

Materials	Volumetric Weight γ/(kN·m^−3^)	Elastic Modulus *E*/GPa	Single-Axis Compressive Strength *σ_c_*/MPa	Poisson Ratio *μ*
Weekly cemented mudstone	23.4	1.96	10.01	0.29
Weakly cemented sandy mudstone	24.1	13.7	11.59	0.20

**Table 5:** Mechanical parameters of similar material samples.

Materials	Volumetric Weight γ/(kN·m^−3^)	Elastic Modulus *E*/GPa	Single-Axis Compressive Strength *σ_c_*/MPa	Poisson Ratio *μ*
Mudstone-like material	25.4	0.098	0.5	0.29
Sandy mudstone-like material	26.1	0.685	0.58	0.20

**Table 6:** Stratum model ratio.

Materials	Rosin–Alcohol Concentration *m*/%	$\frac{IB}{IBS}$	$\frac{I}{IB}$	Hydraulic Oil Content *w*/%
Weakly cemented mudstone	12.5	0.6	0.20	3.5
Weakly cemented sandy mudstone	12.5	0.7	0.35	3.5
Coal	12.5	0.8	0.05	3.5

## Data Availability

The original contributions presented in the study are included in the article, further inquiries can be directed to the corresponding author.

## Nomenclature

**Symbols**	**Implication**
*P*	Prototype materials
*M*	Similar materials
*L*	Geometric dimension
*γ*	Unit weight
*σ*	Stress
*ε*	Strain
*E*	Elastic modulus
*μ*	Poisson’s ratio
*C*	Cohesion
*φ*	Internal friction angle
*σ* *_c_*	Uniaxial compressive strength
*C* *_σ_*	Stress similarity ratio
*C_γ_*	Unit weight similarity ratio
*C_L_*	Geometric similarity ratio
*C* *_δ_*	Displacement similarity ratio
*C_ε_*	Strain similarity ratio
*C_E_*	Elastic modulus similarity ratio
*C_f_*	Friction coefficient similarity ratio
*C_μ_*	Poisson’s ratio similarity ratio
$C_{\sigma_{c}}$	Similarity ratio of uniaxial compressive strength
*C_c_*	Cohesion similarity ratio
*C_t_*	Time similarity ratio
*C_η_*	Viscosity coefficient similarity ratio
*σ*_0_	Creep stress
*E*_0_	Elastic modulus of spring elements
*E*_1_	Elastic modulus of elastomer in Kelvin body
*η*_1_, *η*_2_	Viscosity coefficients
*σ_s_*	Long-term strength
$\dot{\varepsilon }$	Strain rate
